# Aerobic exercise strategies for anxiety and depression among children and adolescents: a systematic review and meta-analysis

**DOI:** 10.3389/fpubh.2025.1555029

**Published:** 2025-07-01

**Authors:** Huishan Song, Sheng Ge, Yuhe Wang, Linghua Ran, Hongyan Zhang

**Affiliations:** ^1^School of Sports Science, Harbin Normal University, Harbin, China; ^2^Central Hospital of Heilongjiang Provincial Prison Administration, Harbin, China; ^3^School of Computer Science and Information Engineering, Harbin Normal University, Harbin, China; ^4^Center for Mental Health Education, Harbin Normal University, Harbin, China

**Keywords:** aerobic exercise (AE), anxiety, depression, children, adolescents

## Abstract

**Objective:**

This study aimed to investigate the effects of exercise intensity, frequency, session duration, and intervention period in aerobic exercise programs on alleviating depression and anxiety symptoms among children and adolescents. The objective of this study is to develop suitable aerobic exercise plans for these individuals.

**Methods:**

All articles published between the database inception year and November 2024 were obtained from PubMed, Scopus, and Web of Science. A meta-analysis was conducted using RevMan 5.4.

**Results:**

The analysis included data from 19 randomized controlled trials involving 2,093 children and adolescents. The findings indicated that aerobic exercise significantly improved anxiety (standardized mean difference [SMD] = −0.32, 95% CI: −0.60, −0.03; *p* < 0.00001) and depression (SMD = −0.64, 95% CI: −0.94, −0.33, *p* < 0.00001). For anxiety symptoms, sessions lasting 60–75 min showed significant effects (SMD = −1.65, 95% CI: −3.25, −0.06, *p* < 0.00001); a frequency of 3–4 sessions/week was most effective (SMD = −0.42, 95% CI: −0.74, −0.10, *p* = 0.001); and interventions exceeding 12 weeks showed significant improvements (SMD = −0.51, 95% CI: −0.89, −0.14, *p* = 0.002). For depression symptoms, sessions lasting 60–75 min produced significant effects (SMD = −0.78, 95% CI: −1.24, −0.33, *p* = 0.001); a frequency of 3–4 sessions/week yielded optimal outcomes (SMD = −0.78, 95% CI: −1.09, −0.46, *p* < 0.00001); and a shorter duration of 5–8 weeks showed significant improvements (SMD = −1.22, 95% CI: −1.79, −0.65, *p* < 0.0001).

**Conclusion:**

For managing anxiety symptoms in children and adolescents, we recommend a high-intensity exercise regimen (60–89% VO_2_ max, 60–75 min/session, 3–4 sessions/week) with an optimal intervention duration of over 12 weeks. For managing depression symptoms, we propose a moderate-to-high-intensity exercise protocol (40–89% VO_2_ max) with the same session duration and frequency, but with a shorter optimal intervention duration of 5–8 weeks.

## 1 Introduction

Aerobic exercise has been identified as a promising non-pharmacological therapy for managing anxiety and depression; however, specific intervention strategies remain unclear. An alarming surge in the prevalence of anxiety and depression among children and adolescents has emerged as a pressing public health concern, with detrimental effects on physical, mental, and social environments (1, 2). Statistics indicate that by the age of 18, approximately 20% of adolescents may experience episodes of depression or anxiety. These disorders often exhibit chronic and recurrent characteristics, with comorbidity rates ranging from 10 to 50% (3). Research has revealed that several factors contribute to the rise in these mental health issues, including rising pressure in academic performance, the influence of social media, and the overall complexity of modern life (4, 5). Moreover, anxiety and depression can lead to physical ailments, such as sleep disorders, obesity, and chronic fatigue, which in turn exacerbate the mental health challenges faced by adolescents (6).

Numerous scholarly studies have meticulously examined the multifaceted advantages of aerobic exercise in adolescents, including its role in fortifying the heart, enhancing bone and muscle health, and mitigating the risk of mental disorders (7, 8). By improving cardiorespiratory fitness, individuals can not only boost their physical performance but also potentially enhance their mental resilience and emotional well-being. This highlights the critical role of cardiorespiratory fitness in the context of exercise to combat anxiety and depression. Moreover, as a key aspect of physical activity, aerobic exercise has been suggested as an effective approach to tackle physical manifestations and mental health challenges, serving as a valuable adjunctive therapy for anxiety and depression (9). For instance, a meta-analysis conducted by Larun et al. synthesized data from multiple randomized controlled trials (1). They found that exercise interventions significantly reduced the symptoms of anxiety and depression in adolescents aged 12–18 years. The effect sizes were moderate to large, indicating a substantial beneficial impact. Another longitudinal study by Werner-Seidler et al. assessed a cohort of children aged 8–13 years (3). The researchers observed that those who participated in regular school-based physical activity programs had lower levels of anxiety and depression symptoms over a 2-year period compared to their sedentary counterparts. Therefore, implementing comprehensive programs that promote regular physical activity can play a pivotal role in cultivating healthier and more balanced lifestyles among youth, ultimately leading to long-term improvements in both mental health and overall life satisfaction.

Although numerous studies have explored the impact of exercise on health, there are still significant gaps in understanding the optimal parameters for aerobic training, especially in specific populations, such as children and adolescents. The optimal levels of intensity, frequency, session duration, and intervention period of exercise have yet to be established. Our research systematically explores these key parameters and aims to fill a critical gap in the current academic literature. Consequently, the central research inquiry is to assess the effectiveness of the intensity, frequency, session length, and intervention period of the aerobic exercise program in alleviating the symptoms of depression and anxiety, thereby providing a theoretical foundation for creating individualized aerobic training regimens for children and adolescents.

## 2 Materials and methods

### 2.1 Search strategy

This systematic review adhered to the PRISMA (Preferred Reporting Items for Systematic reviews and Meta-Analyses) guidelines for reporting meta-analyses. This study was also pre-registered in the international database of prospectively registered systematic reviews in health and social care (10) (PROSPERO CRD42024618219).

We conducted a search across three electronic databases, namely PubMed, Scopus, and Web of Science, to identify relevant articles published between the database’s inception year and November 2024. The terms utilized for electronic searches included (adolescent OR teenager OR children OR student) AND (sport OR “aerobic exercise” OR “aerobic training”) AND (depressive OR depression OR anxiety OR “mental health” OR “emotional symptom” OR anxious). Supplementary File S1 outlines the particular search tactics for the three electronic databases. In addition, we manually searched the reference lists of the relevant systematic reviews to identify more eligible studies.

### 2.2 Selection criteria

The title and abstract of the article were scrutinized by two authors (H. S. Song and S. GE), with a final evaluation and decision made by a third author (L. H. RAN).

Research studies were included if they met the following criteria: (1) involved children and adolescents aged 6–19 years; (2) examined the effects of aerobic exercise; (3) included a control group (such as usual care, health education, or no treatment); (4) assessed outcomes related to anxiety and depression; and (5) were designed as clinical randomized controlled trials (RCTs).

Research studies were excluded if they met any of the following criteria: (1) a repeated publication, (2) lack of a control group, (3) omission of the entire text, or (4) absence of data or unclear reporting of data for analysis.

### 2.3 Data extraction and subgroup analysis

Information was gathered from the selected research studies, which encompassed elements including the primary author’s name, publication year, size of the sample, participant information (age and numbers), intervention design (both experimental and control groups), treatment specifics (intensity, frequency, session duration, and intervention period), and outcome measures. Literature screening was performed by two researchers (H. S. Song and Y. H. Wang), and the results of the two screenings were compared. In case of disagreement, a third researcher (L. H. Ran) decided on inclusion of the studies.

In terms of subgroup analyses, we conducted two distinct subgroup analyses for anxiety and depression outcomes according to the intensity, frequency, session duration, and intervention period of the exercise intervention in the experimental group. The aim of this study is to establish the best fitness regimen for children and adolescents who suffer from anxiety and depression.

### 2.4 Risk of bias and quality assessment

We evaluated the potential for bias in each of the included studies using the guidelines detailed in the Cochrane Handbook and applying the PEDro scale (11). The evaluation of quality encompassed the development of random sequences, methods to conceal allocation, blinding of both researchers and participants, masking of study findings, etc. For every requirement that was satisfied, one point was awarded. The evaluation scale extended from 0 to 10, where ratings of 9 to 10 signified outstanding quality, scores between 6 and 8 represented good quality, ratings of 4 to 5 indicated acceptable quality, and any score below 4 denoted subpar quality (12). Additionally, risk was assessed according to the following criteria: unclear, low, and high. Two researchers (H. S. Song and H. Y. Zhang) independently evaluated the quality of the literature and then cross-checked it with a third researcher (L. H. Ran) to determine the risk level in ambiguous literature.

### 2.5 Statistical analysis

The software RevMan 5.4 for Windows was used to conduct the meta-analyses. Standardized mean differences (SMD) were selected as helpful indicators, and the variance was presented as a 95% confidence interval (CI). If there was no discernible difference according to the heterogeneity test, a fixed-effects model was used. Otherwise, a random-effects model was used. Heterogeneity was evaluated using the I^2^ statistic. Heterogeneity was considered high when I^2^ was greater than 75%. In-depth subgroup analyses were performed according to various comparison types. We conducted a sensitivity assessment by individually excluding each study to assess the robustness of the findings. Additionally, we assessed publication bias by visually examining funnel plots and employing statistical methods, provided that a minimum of 10 research projects were incorporated into the meta-analysis in accordance with the guidelines set by the Cochrane Collaboration.

## 3 Results

### 3.1 Search and screening outcomes

**Figure 1:**
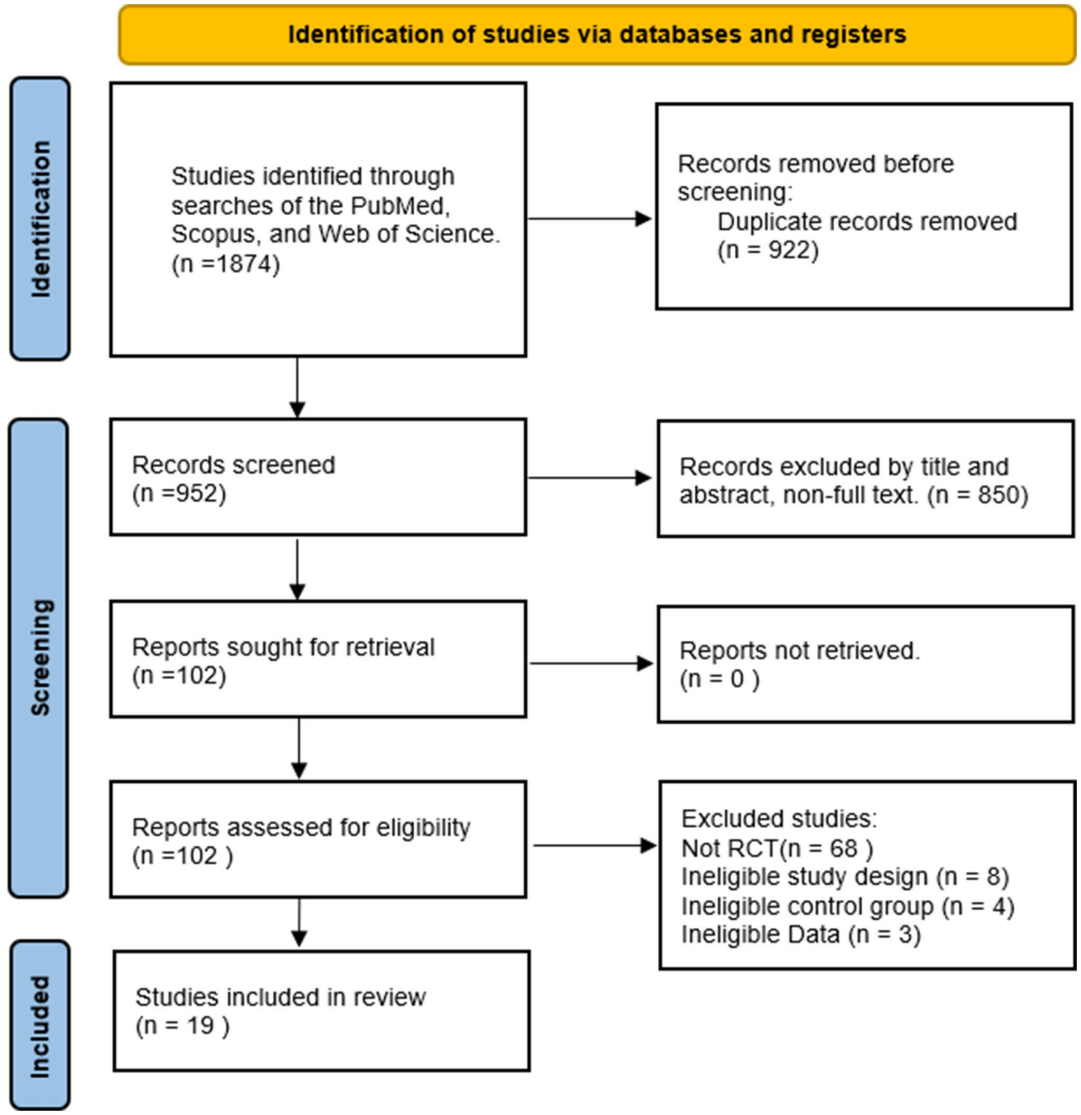
Flowchart based on PRISMA standards.

The selection process is shown in Figure 1. Studies were identified through searches of PubMed, Scopus, and Web of Science. A total of 1874 records were retrieved. Inclusion and exclusion criteria were applied to screen titles and abstracts, resulting in the exclusion of 1,772 studies that did not meet eligibility requirements. Full texts of the remaining 102 studies were assessed, and 19 studies were ultimately included in the meta-analysis.

### 3.2 Research characteristics

Table 1 summarizes the characteristics of the 19 studies (13–31), detailing publication dates, sample sizes, and demographics of the populations examined by age. It also includes the design of treatment interventions concerning mode, intensity, duration, and frequency, along with information about the control groups and the outcome measures assessed. This analysis encompassed findings from 19 randomized controlled trials involving 2093 children and adolescents. The 19 studies were broadly categorized into aerobic exercises of different types based on their exercise content. Each exercise session varied in length from 15 to 75 min, and the intervention period ranged from 5 to 24 weeks. Additionally, the frequency of exercise interventions varied from 1 to 7 times per week. The measured outcomes mainly consisted of depression and anxiety. The specifics of this study are shown in Table 1.

**Table 1:** The character of included studies.

No.	Included studies	Age	Sample Size (T/C)	Intervention			Control group	Outcome measure
				Training movement	Intensity	Frequency; duration		
1	Bao 2015 (13)	14.74 ± 0.66	80/80	Tai Chi	NR	60 min/time 5 times/week 12 months	No exercise	Anxiety: PHCSCS SP: SPPC
2	Dabidy 2011 (14)	T:16.91 ± 1.03 C: 16.83 ± 0.82	12/12	Pool walking exercise	60–70% HRmax	15 min/time 3 times/week 6 weeks	No exercise	Depression: Ham-D
3	Fidelix 2019 (15)	T:14.64 ± 1.14 C: 14.64 ± 1.18	31/31	High intensity exercise	80–90%VO_2_ max	60 min/time 3 times/week 24 weeks	Routine exercises	Anxiety: STAI Depression: BDI; SP: RSES
4	Goldfield 2015 (16)	T: 15.5 ± 1.4 C: 15.6 ± 1.3	75/76	Treadmills, elliptical machines	65–85% HRmax	65 min/time 4times/week; 24 weeks	No exercise	Anxiety: BRUMS-Tension; Depression: BRUMS-Depression; SP: GSW
5	Jelalian 2011 (17)	14.20 ± 0.93	45/44	Aerobic exercise	NR	1 times/week 16 weeks	Peer enhanced adventure therapy	Anxiety: SAS-A SP: GSW; BMI
6	Jeong 2005 (18)	16	20/20	Dancing	NR	45 min/time 3 times/week 12 weeks	Routine exercises	Anxiety: SCL-90 Depression: SCL-90
7	Luo 2021 (19)	T: 13.83 ± 0.39 C: 13.96 ± 0.21	23/34	Aerobic exercise	RPE:11–14	30 min/time 4times/week; 12 weeks	No exercise	Anxiety: SAS Depression: SDS
8	Melnyk 2009 (20)	15.5 ± 0.63	12/7	Kickball, walking	NR	15–20 min/time 2–3 times/week 9 weeks	No exercise	Anxiety: BDI-anxiety Depression: BDI-depression
9	Nazari 2020 (21)	T: 11.22 ± 1.9 C: 11.00 ± 2.67	20/20	Aerobic exercise	50–75% HRmax	60 min/time 3 times/week 16 weeks	No exercise	Anxiety: RCMAS
10	Petty 2009 (22)	AE(a): 9.3 ± 0.9 AE(b): 9.3 ± 1 C: 9.4 ± 1.2	AE(a)/C: 68/68 AE(b)/C: 70/68	AE(a): HIIT (low dose) AE(b): HIIT (high dose)	160–175bmp	20–40 min /time 6 times/week; 13 ± 1.6 weeks	No exercise	Depression: RCDS; SP: GSW; BMI
11	Philippot 2022 (23)	T: 15.2 ± 1.5 C: 15.5 ± 1.77	20/20	Aerobic exercise	40–59% HRmax	60 min/time 4 times/week 5 weeks	Social relaxation activity	Anxiety: HADS-A Depression: HADS-D
12	Roh 2018 (24)	T: 11.53 ± 0.64 C: 11.40 ± 0.63	15/15	Taekwondo	NR	60 min/time 1 times/week 16 weeks	Baseline activity levels	Anxiety: HADS-A Depression: HADS-D
13	Romero 2020 (25)	10.02 ± 0.79	54/51	Specific exercises and pre-sport games	NR	50 min/time 2 times/week; 20 weeks	No exercise	Anxiety: CMAS-R; Depression: RCDS BMI
14	Roth 1987 (26)	18.9 ± 1.3	18/18	Aerobic exercise	90% HRmax	30 min/time 3 times/week; 11 weeks	No exercise	Anxiety: STAI Depression: BDI
15	Silva 2020 (27)	11–14	10/10	Swimming training	NR	45 min/time 2 times/week; 8 week	Routine exercises	Anxiety: BAI Depression: CDI
16	Talakoub 2012 (28)	14–20	32/32	Aerobic exercise	50–70% HRmax	60 min/time 3 times/week; 6 week	No exercise	Anxiety: SCL-90 Depression: SCL-90
17	Wagener 2012 (29)	14 ± 1.66	21/20	Group dance-based exergame exercise	75% HRmax	75 min/time 3 times/week; 10 weeks	Baseline activity levels	BASC-2; SP: PCS; BMI
18	Weintraub 2008 (30)	T: 9.50 ± 0.58 C: 10.34 ± 0.84	9/12	Soccer program	NR	60 min/times 4 times/week 12 weeks	Health education	Depression: CDI; SP: RSES; BMI
19	Williams 2019 (31)	9.7 ± 0.9	90/85	Vigorous aerobic activities and games	NR	40 min /time; 7 times/week; 24 weeks	½ hr. of supervised homework	Depression: CDI; SP: GSW

AE, aerobic exercise; HIIT, high-intensity interval training; NR, not recorded; STAI, Spielberger State–Trait Anxiety Inventory; GSW, global self-worth; RCDS, Reynolds Child Depression Scale; SP, self perception; SPPC, self-perception profile for children; CMAS-R, Manifest Anxiety Scale in Children-Revised; PCS, Perceived Competence Scale; BASC-2, Behavior Assessment System for Children-2; PRS-A, Parent Rating Scales-Adolescent version; SRP-A, Adolescent Self-Report Scales; RSES, Rosenberg Self-esteem Scale; CDI, Children’s Depression Inventory; QOL, Quality of life score; PedsQL, Pediatric Quality of Life Inventory; PAES, Pediatric Anger Expression Scale; HLBS, Healthy Lifestyle Beliefs Scale; BDI, The Beck Depression Inventory; PEQ, Peer Experiences Questionnaire; SAS-A, Social Anxiety Scale for Adolescents; BRUMS, Brunell Mood Scale.

### 3.3 Study quality and publication bias

**Figure 2:**
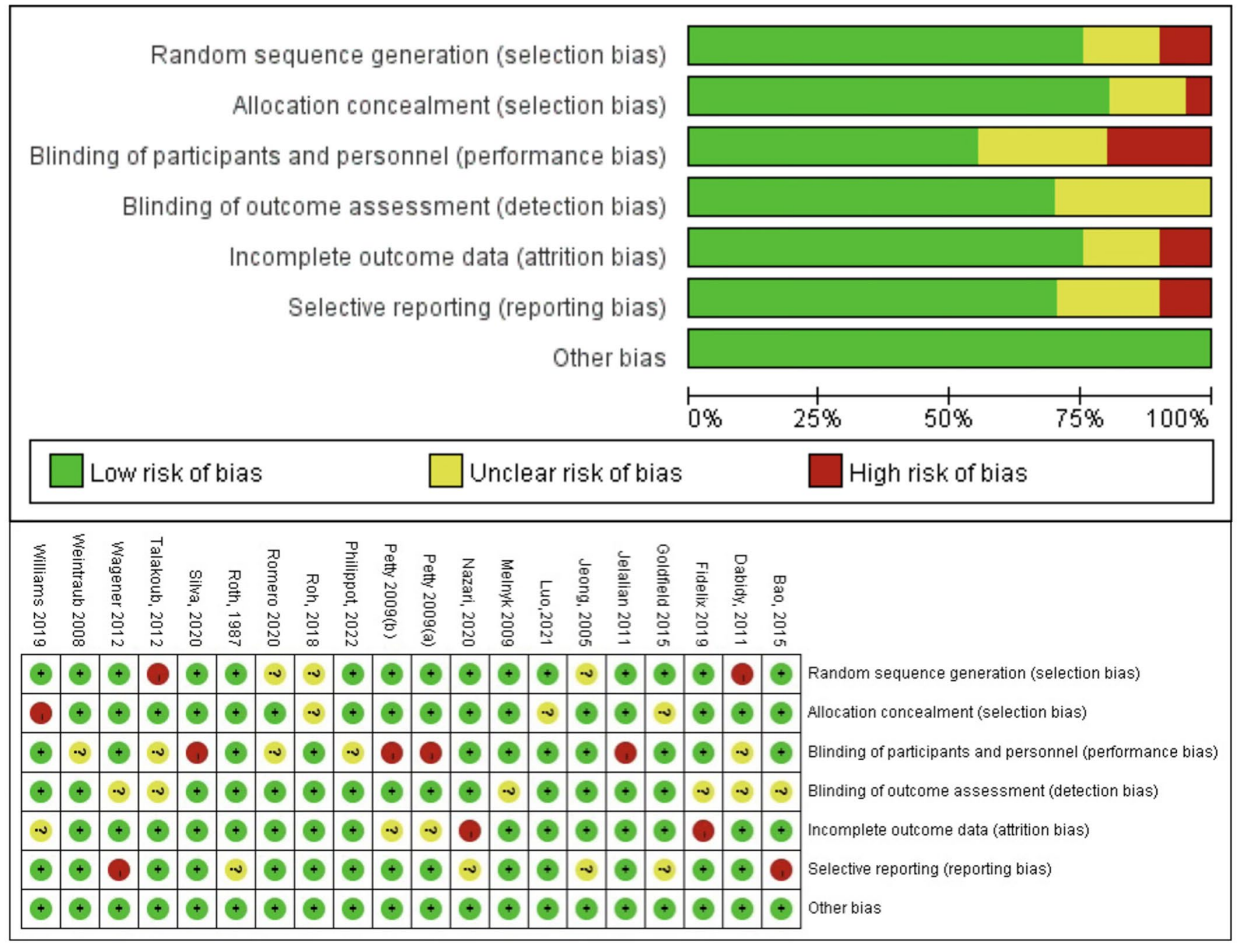
Evaluation of bias risk based on Cochrane collaboration guidelines.

**Figure 3:**
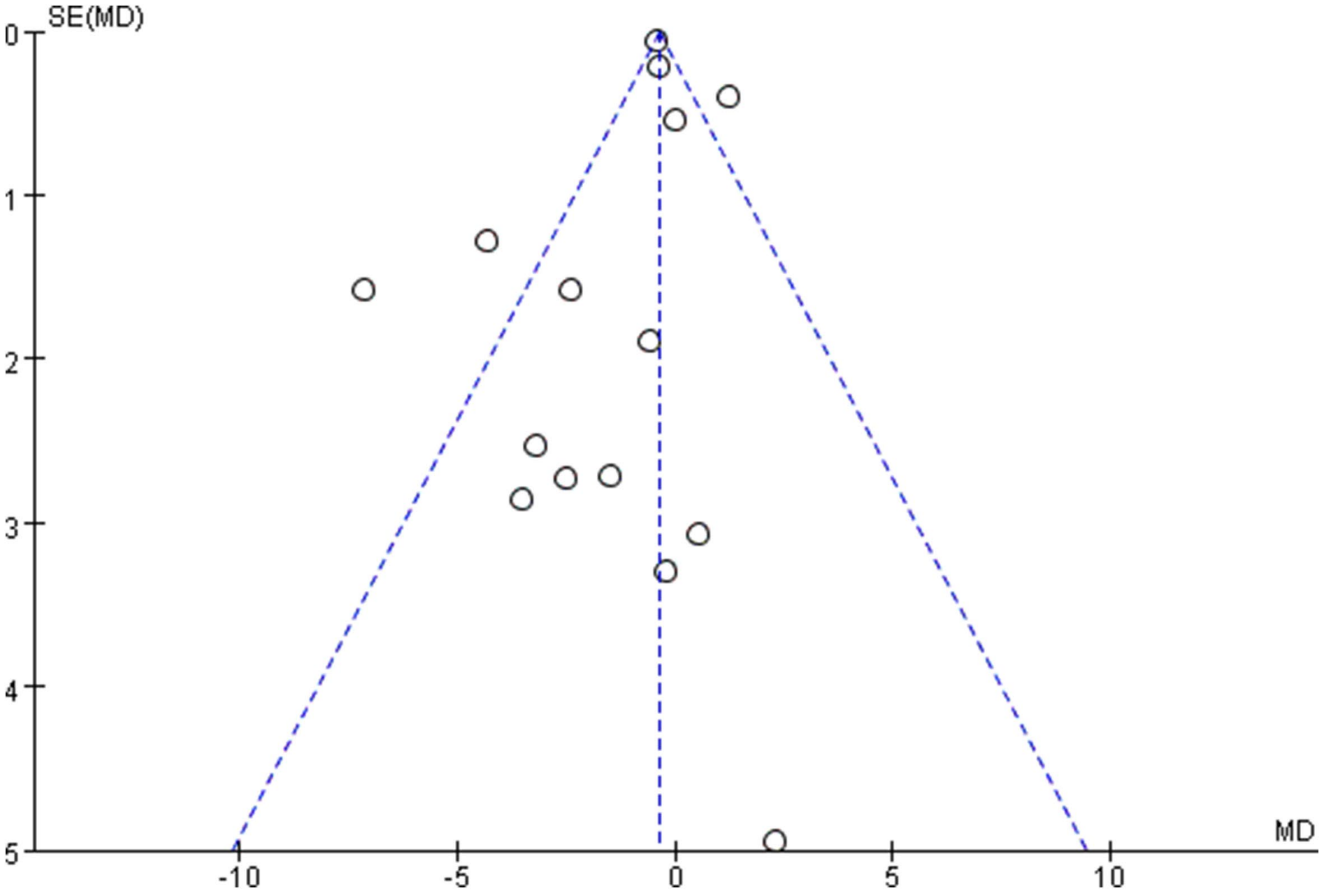
Funnel plot of anxiety.

**Figure 4:**
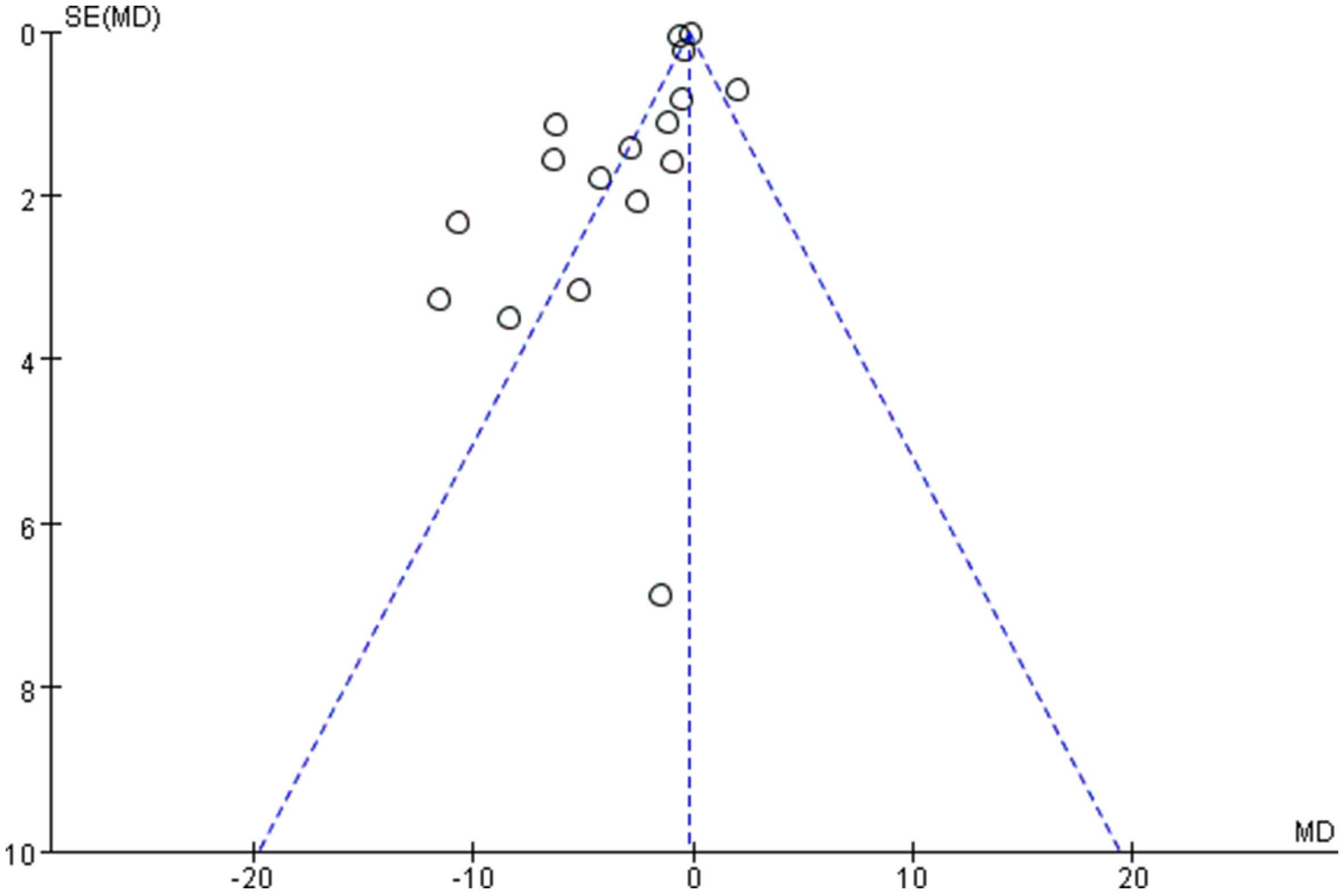
Funnel plot of depression.

The overall quality of the enrolled publications was relatively high in the quality evaluation of the RCTs. Supplementary File S2 displays each study’s PEDro scores for quality evaluation. Figure 2 shows the specifics regarding the included studies’ risk of bias. The PEDro scores of most studies were > 6, and only two studies had a score of 5. The assessment of bias showed that a relatively small percentage of studies were at high risk, with the majority falling into the “unclear risk of bias” category. There were no notable biases in the general results of this study. The risk assessment was then entered separately into Review Manager (RevMan 5.4) for each trial, resulting in a risk-of-bias summary that was provided together with the meta-analysis findings. According to the funnel diagram, there was no evidence of publication bias among any of the treatment research projects, which included evaluations of anxiety (Figure 3) or depression (Figure 4).

### 3.4 Outcomes

#### 3.4.1 Anxiety

**Figure 5:**
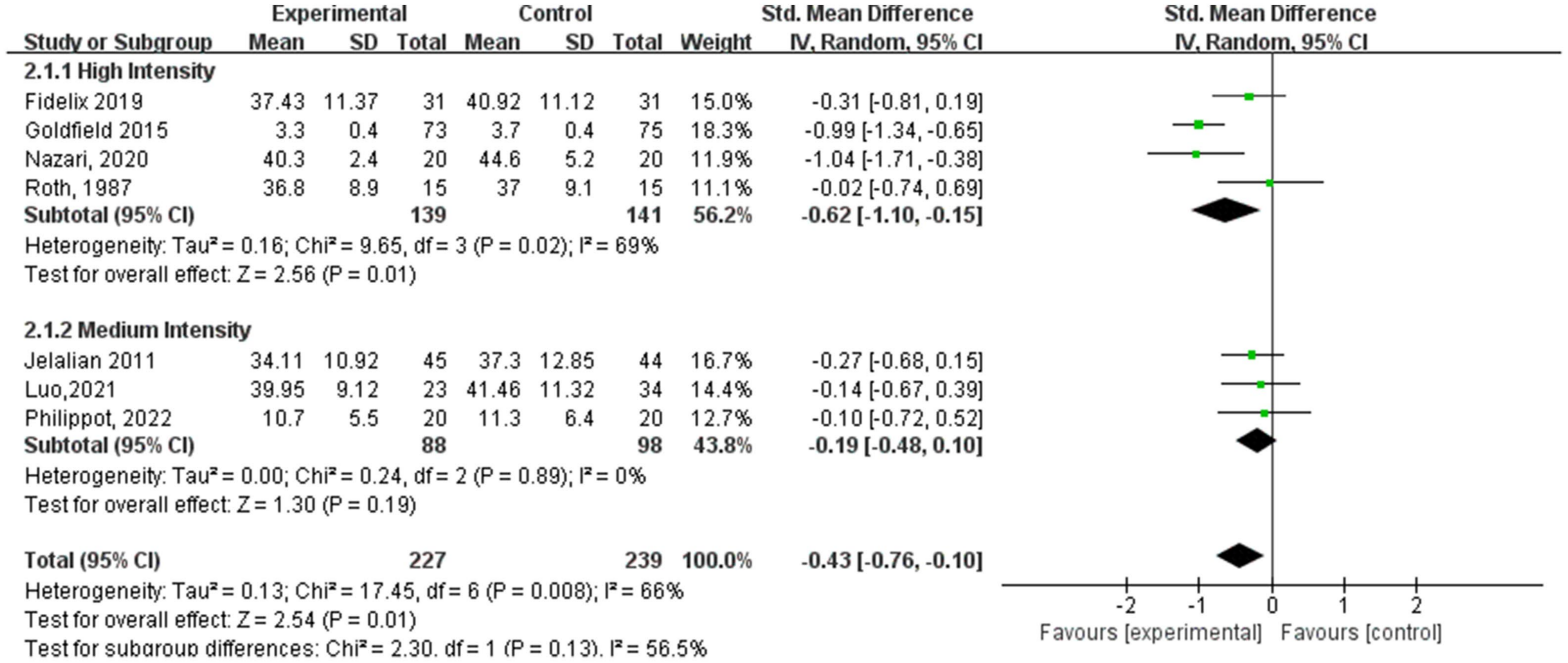
Anxiety results for subgroup analysis based on exercise intensity.

**Figure 6:**
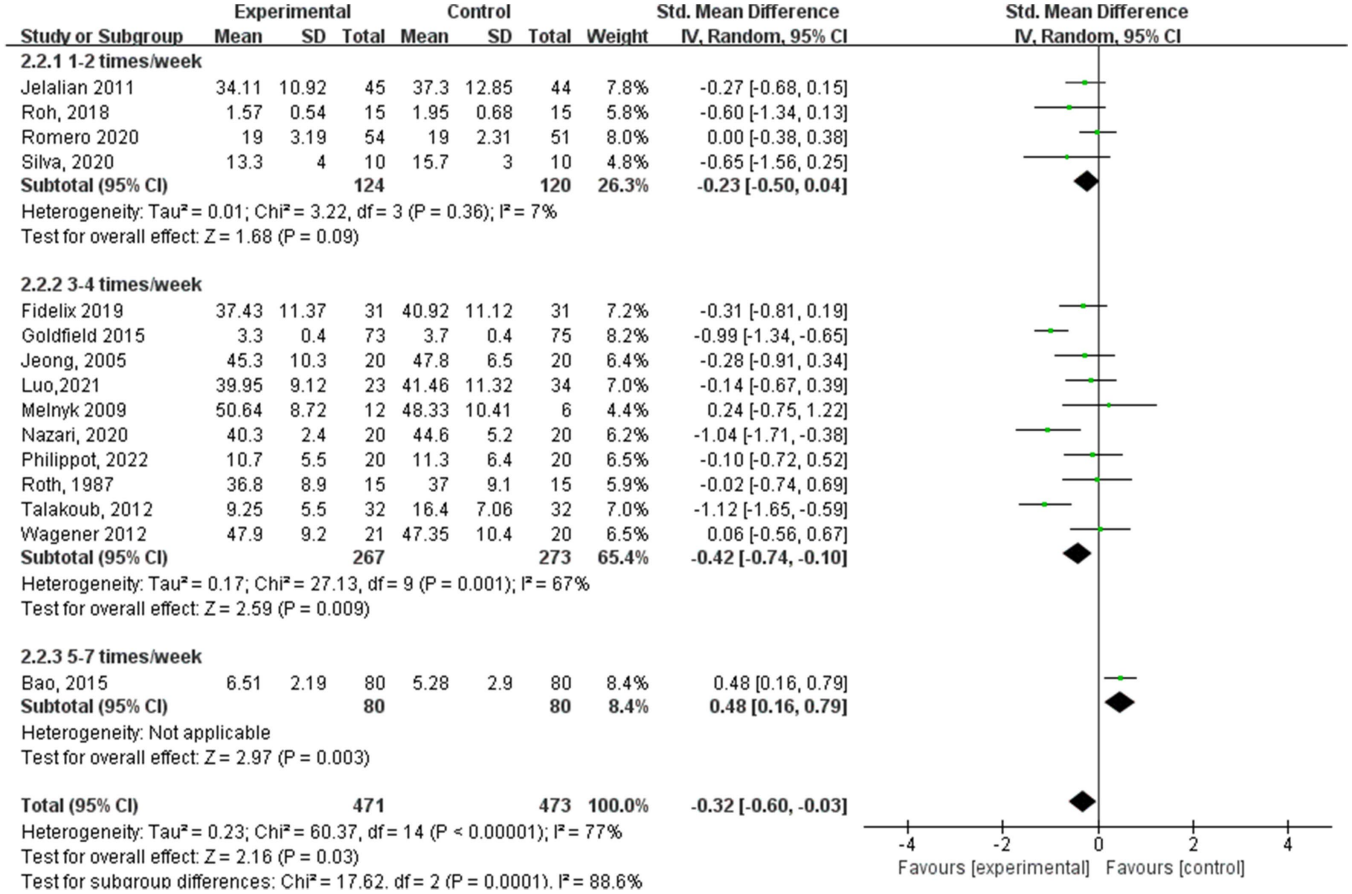
Anxiety results for subgroup analysis based on exercise frequency.

**Figure 7:**
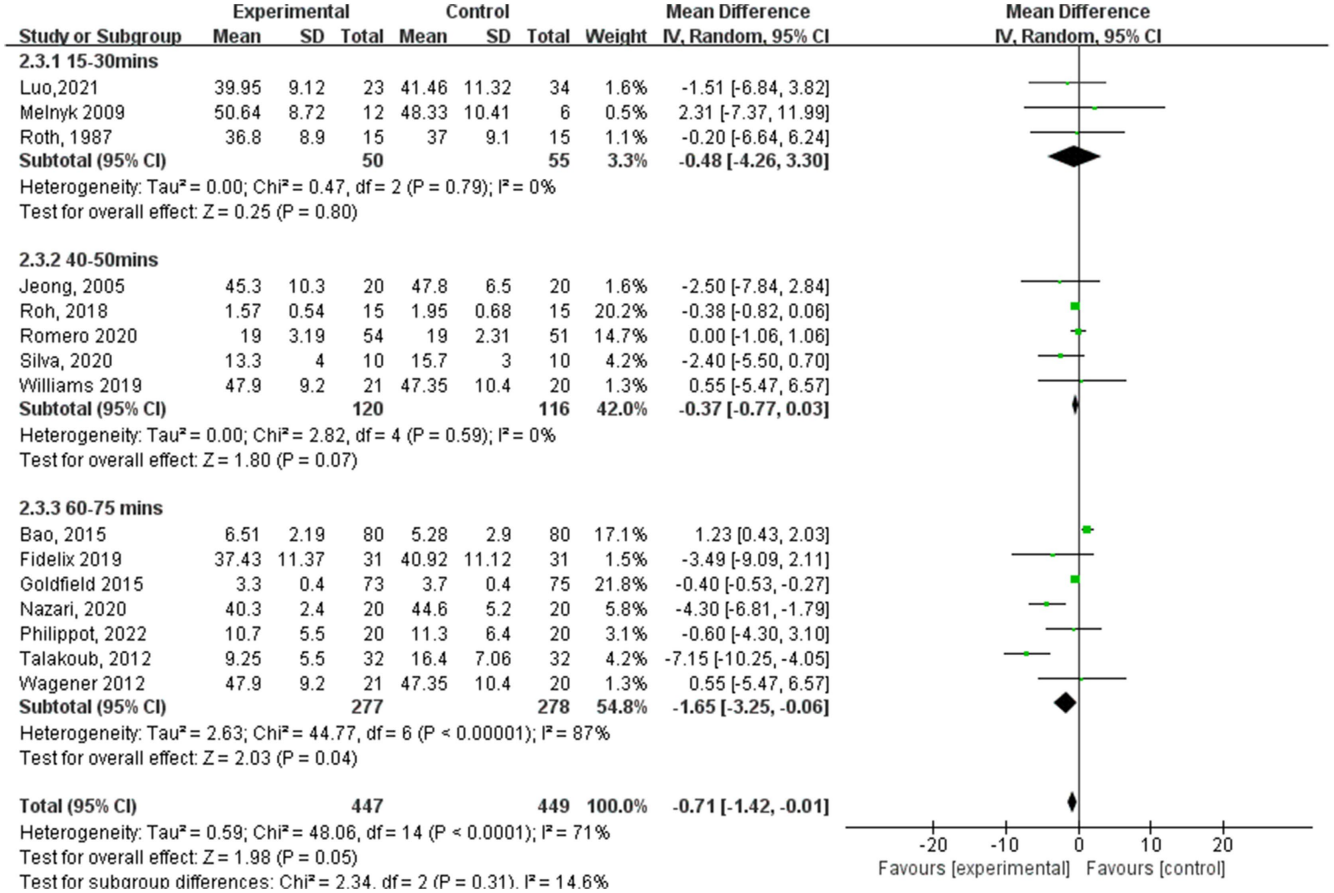
Anxiety results for subgroup analysis based on exercise session duration.

**Figure 8:**
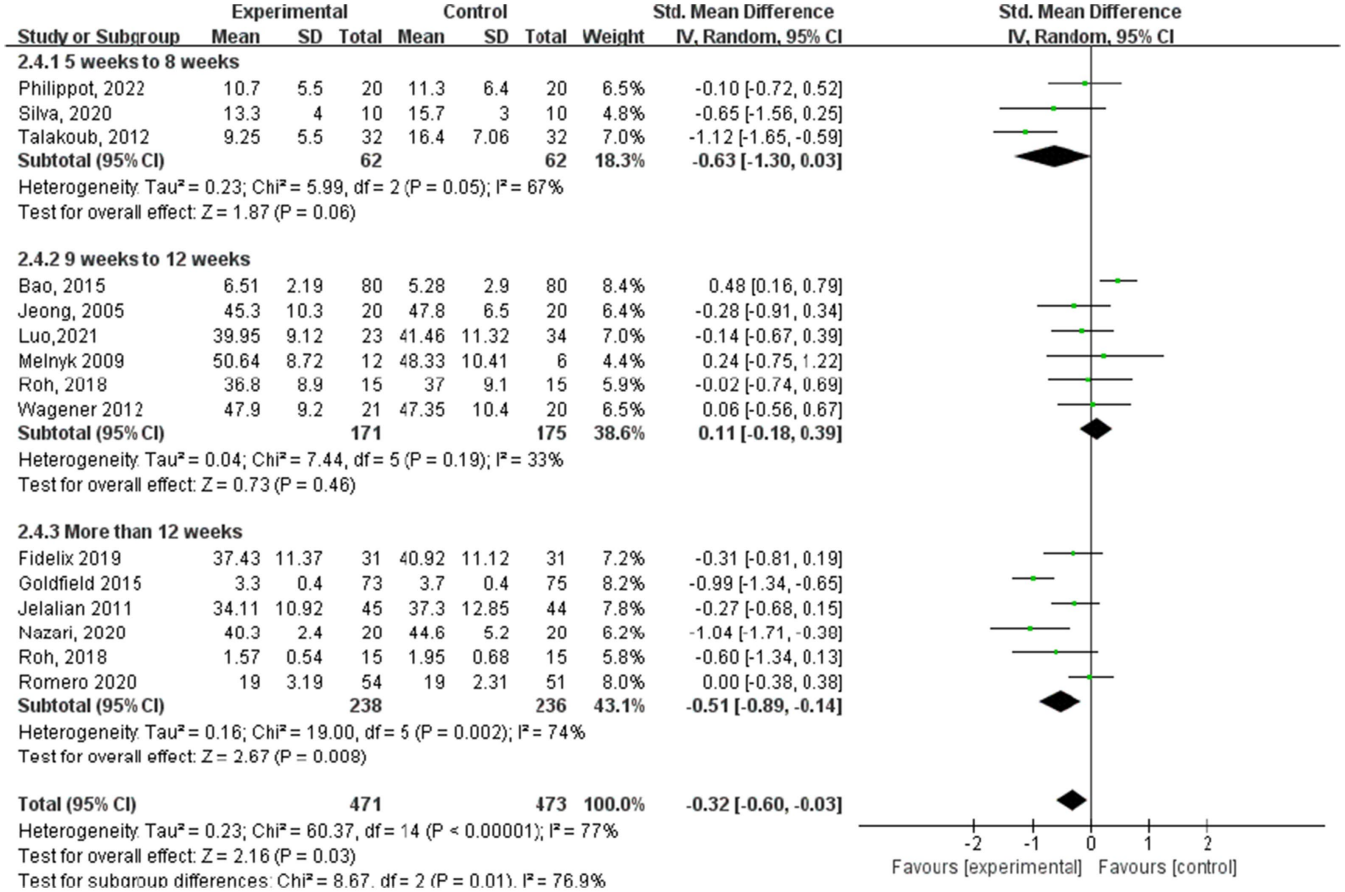
Anxiety results for subgroup analysis based on exercise intervention period.

Figures 5–8 show a forest plot of a subgroup analysis of 19 studies based on different aerobic exercise treatments (intensity, frequency, session duration, and intervention period), using anxiety as the outcome for children and adolescents. The anxiety levels in the aerobic exercise training group were significantly lower than those in the control group when all exercise modes were considered (SMD = −0.32, 95% CI: −0.60 to −0.03, *p* < 0.00001, I^2^ = 77%).

In the subgroup analysis of aerobic exercise intensity (Figure 5), 4 subjects conducted high-intensity training (60–89% of VO_2_ max), and 3 subjects conducted moderate-intensity training (40–59% of VO_2_ max). According to aerobic exercise intensity, the study participants who participated in interventions for high-intensity exercise had the best experimental effect (SMD = −0.62, 95% CI: −1.10 to −0.14, *p* = 0.01, I^2^ = 69%), whereas those who participated in medium-intensity aerobic exercise did not exhibit significant effects (SMD = −0.19, 95% CI: −0.48 to 0.10, *p* = 0.19, I^2^ = 0%).

Within the subgroup analysis of aerobic exercise regimen frequency (Figure 6), 4 studies (17, 24, 25, 27) reported aerobic exercise at 1–2 sessions per week, 10 studies (15–17, 19–21, 23, 26, 28, 29) at 3–4 sessions per week, and 1 study (13) at 5–7 sessions per week. In terms of anxiety results based on aerobic exercise frequency, the study group that engaged in interventions 3–4 times a week showed the best experimental effect (SMD = −0.42, 95% CI: −0.74 to −0.10, *p* = 0.001, I^2^ = 67%), whereas those engaged in interventions 1–2 times a week did not show significant effects (SMD = −0.23, 95% CI: −0.50 to 0.04, *p* = 0.36, I^2^ = 7%).

In the subgroup analysis of aerobic exercise session duration (Figure 7), 3 studies (19, 20, 26) reported aerobic exercise at 15–30 min per session, 5 studies (18, 24, 25, 27, 31) at 45–50 min per session, and 7 studies (13, 15, 16, 21, 23, 28, 29) at 60–75 min per session. According to aerobic exercise session duration, the study participants who participated in interventions for 60–75 min had the best experimental effect (SMD = −1.65, 95% CI: −3.25 to −0.06, *p* < 0.00001, I^2^ = 87%), whereas those who participated for 15–30 min and 40–50 min did not exhibit significant effects (SMD = −0.48, 95% CI: −4.26 to 3.30, *p* = 0.79, I^2^ = 0%; SMD = −0.37, 95% CI: −0.77 to 0.03, *p* = 0.59, I^2^ = 0%).

In the subgroup analysis of the aerobic exercise intervention period (Figure 8), 3 studies (23, 27, 28) indicated periods of 5–8 weeks, whereas 6 studies (13, 18–20, 24, 29) reported periods ranging from 9 to 12 weeks. Additionally, another 6 studies (15–17, 21, 24, 25) documented aerobic exercise lasting over 12 weeks. Regarding anxiety outcomes based on the intervention period of aerobic exercise, the study group that engaged in interventions for more than 12 weeks showed the best experimental effect (SMD = −0.51, 95% CI: −0.89 to −0.14, *p* = 0.002, I^2^ = 74%), whereas those groups with 5–8 weeks and 9–12 weeks did not show significant effects (SMD = −0.63, 95% CI: −1.30 to 0.03, *p* = 0.05, I^2^ = 67%; SMD = 0.11, 95% CI: −0.18 to 0.39, *p* = 0.19, I^2^ = 33%).

#### 3.4.2 Depression

**Figure 9:**
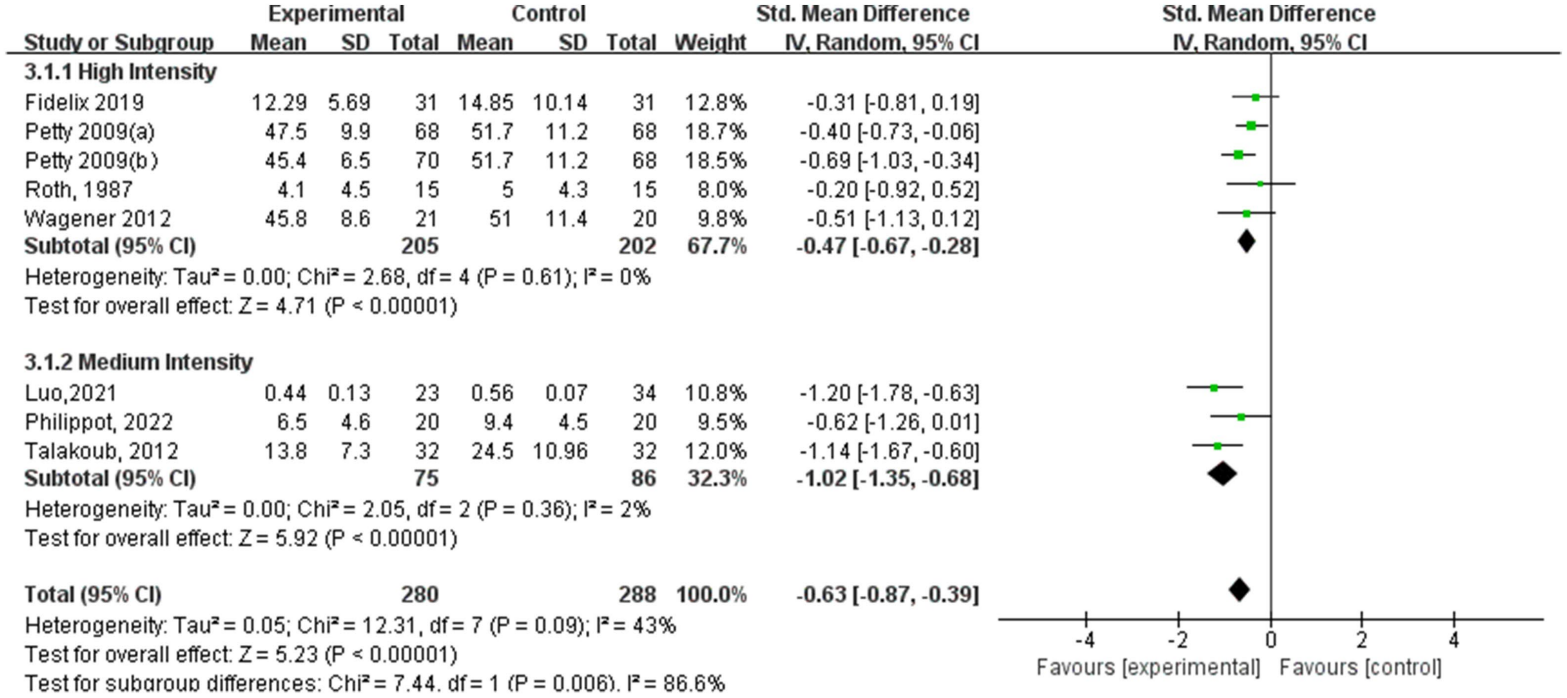
Depression results of subgroup analysis based on exercise intensity.

**Figure 10:**
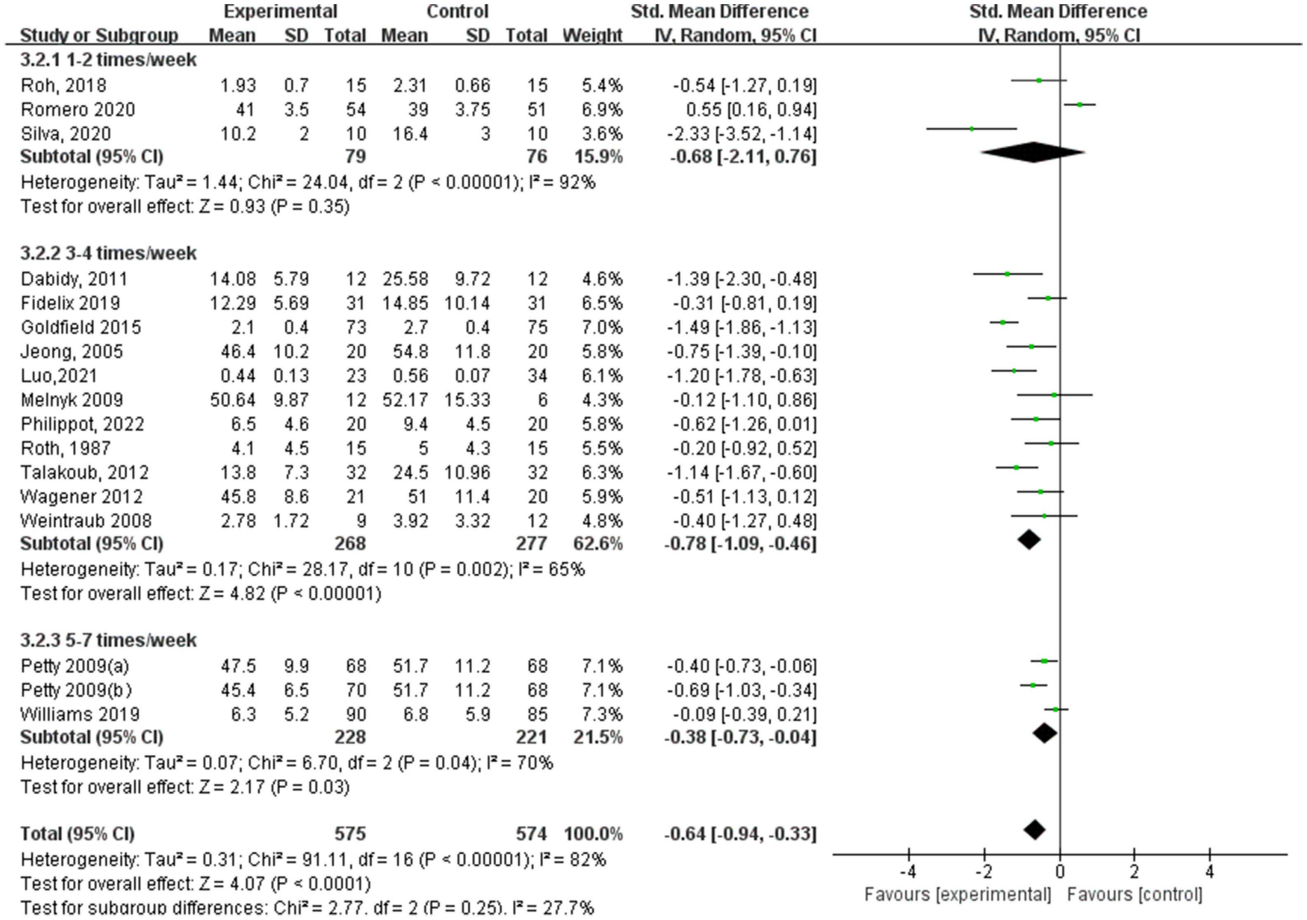
Depression results of subgroup analysis based on exercise frequency.

**Figure 11:**
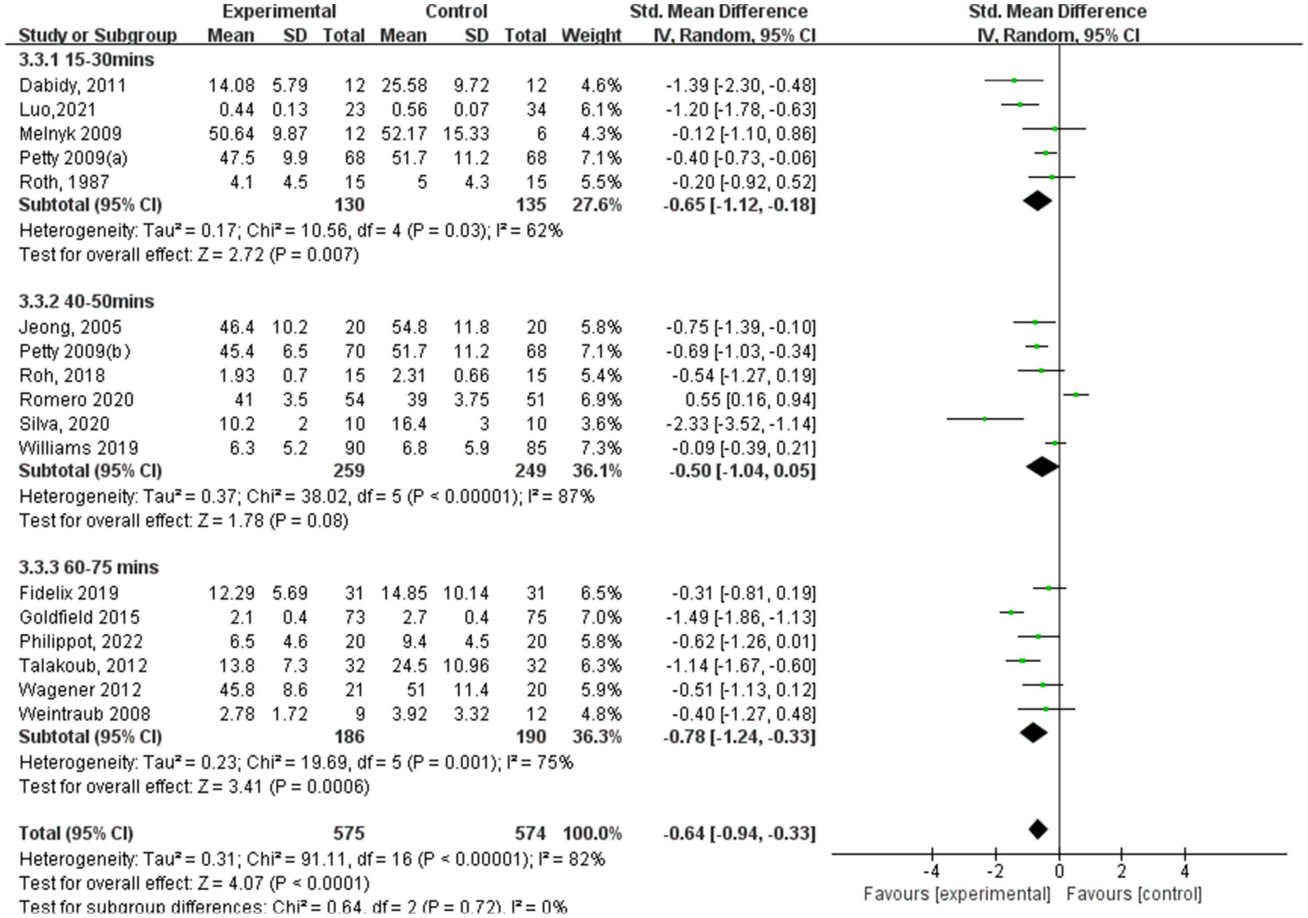
Depression results of subgroup analysis based on exercise session duration.

**Figure 12:**
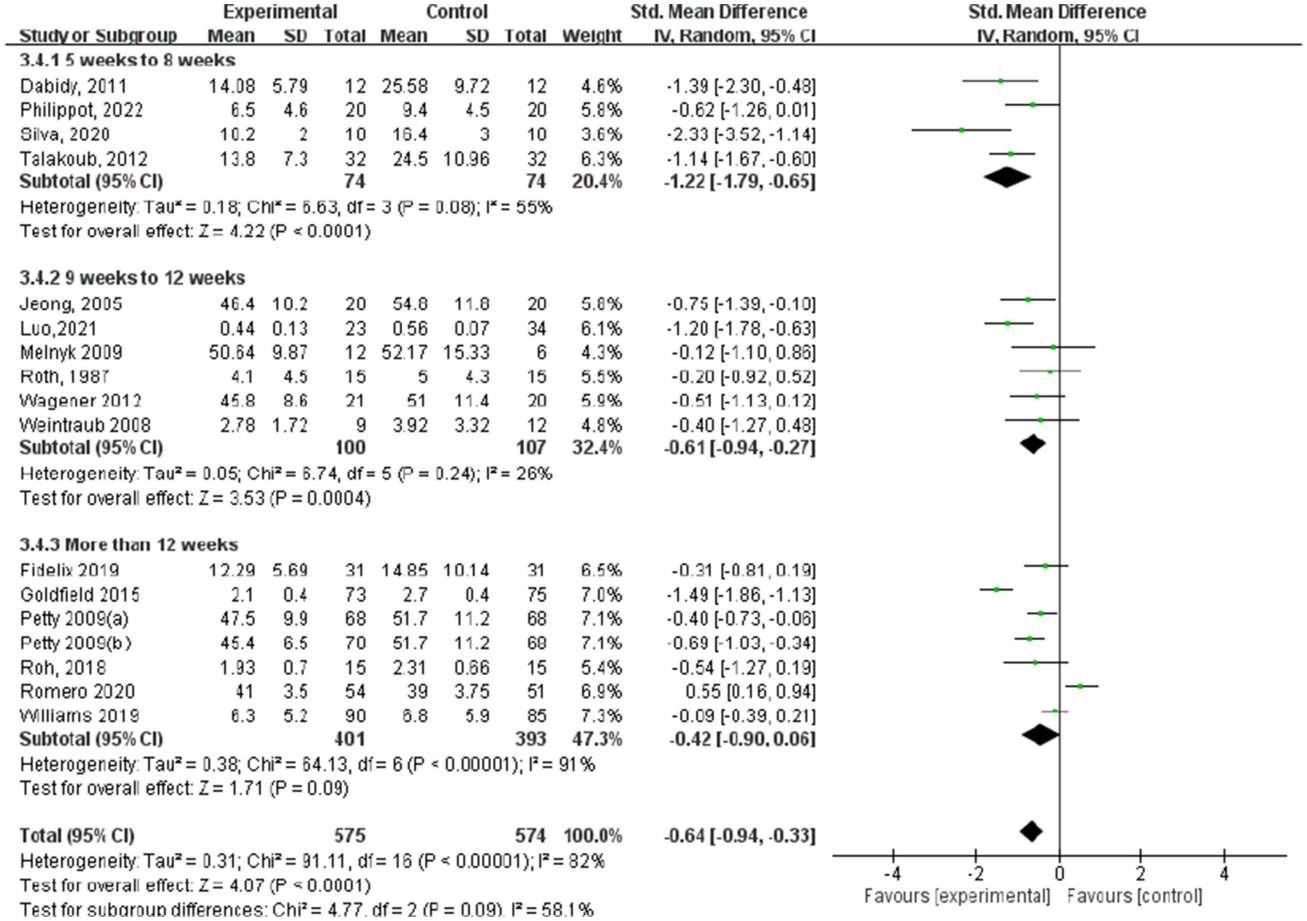
Depression results of subgroup analysis based on exercise intervention period.

Figures 9–12 are forest plots showing the subgroup analysis of the 19 studies based on aerobic exercise therapies, with depression as the outcome in children and adolescents. The depressive outcomes in the aerobic exercise group were notably reduced when compared to the control group, considering that all forms of exercise were pooled together (SMD = −0.64, 95% CI: −0.94 to −0.33, *p* < 0.00001, I^2^ = 82%).

Among the subgroup analysis of aerobic exercise intensity (Figure 9), five studies conducted high-intensity training at 60–89% of VO_2_ max, and three studies conducted moderate-intensity training at 40–59% of VO_2_ max. Both high- and moderate-intensity aerobic exercise interventions demonstrated significant effects in alleviating depression symptoms. The high-intensity training group showed a substantial impact (SMD = −0.47, 95% CI: −0.67 to −0.28, *p* < 0.00001, I^2^ = 0%), indicating a strong association between high-intensity exercise and reduced depression symptoms. Similarly, the moderate-intensity aerobic exercise group also achieved significant results (SMD = −1.02, 95% CI: −1.35 to −0.68, *p* < 0.00001, I^2^ = 2%), suggesting that moderate-intensity exercise is also an effective approach for improving depressive conditions.

In terms of depression results based on aerobic exercise frequency (Figure 10), the study group that engaged in interventions 3–4 times a week showed the best experimental effect (SMD = −0.78, 95% CI: −1.09 to −0.46, *p* < 0.00001, I^2^ = 65%), followed by the experimental group with 5–7 times a week (SMD = −0.38, 95% CI: −0.73 to −0.04, *p* = 0.03, I^2^ = 70%). Additionally, those with 1–2 times a week did not show significant effects (SMD = −0.68, 95% CI: −2.11 to 0.76, *p* = 0.35, I^2^ = 92%).

According to aerobic exercise session duration (Figure 11), the study participants who participated in interventions for 60–75 min had the best experimental effect (SMD = −0.78, 95% CI: −1.24 to −0.33, *p* = 0.001, I^2^ = 75%), followed by the experimental group with 15–30 min (SMD = −0.65, 95% CI: −1.12 to −0.18, *p* = 0.007, I^2^ = 62%). Additionally, those who participated for 40–50 min did not exhibit significant effects (SMD = −0.50, 95% CI: −1.04 to 0.05, *p* = 0.08, I^2^ = 87%).

Regarding depression outcomes based on the intervention period of aerobic exercise (Figure 12), the study group that engaged in interventions of 5–8 weeks showed the best experimental effect (SMD = −1.22, 95% CI: −1.79 to −0.65, *p* < 0.0001, I^2^ = 55%), whereas those with more than 12 weeks did not show significant effects (SMD = −0.42, 95% CI: −0.90 to 0.06, *p* = 0.09).

### 3.5 Sensitivity analysis

**Figure 13:**
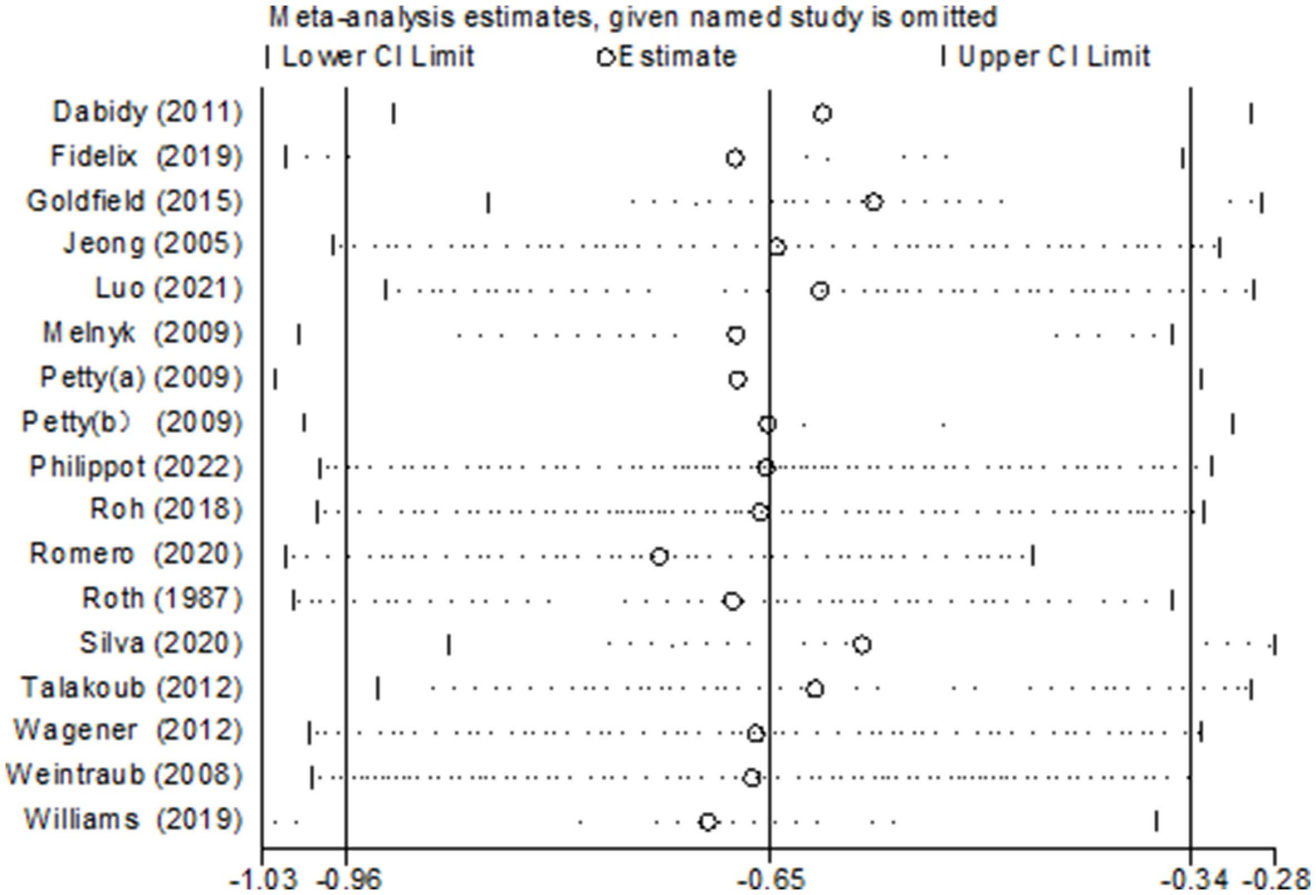
Sensitivity analysis of the relationship between exercise and depression.

To investigate the potential sources of heterogeneity, sensitivity analyses were conducted through the sequential exclusion of individual studies (Figure 13). Consistent with the primary analysis, the removal of any single study demonstrated a minimal influence on the pooled estimates, indicating the robustness of the combined effect values obtained in this research.

## 4 Discussion

The outcomes of this meta-analysis suggest that aerobic training can significantly improve anxiety and depression in children and adolescents, consistent with previous research findings (32–36). Neuroimaging studies demonstrate that regular aerobic exercise enhances prefrontal cortex volume and top-down emotional regulation in adolescents, which may mediate its positive effects on emotional adjustment (37). Aerobic exercise modulates physiological stress and the hypothalamic–pituitary–adrenal axis, potentially explaining its anxiolytic effects. It reduces hypothalamic neuropeptide expression and decreases sensitivity to stressors. Studies have shown that aerobic exercise suppresses CRH expression, thereby lowering ACTH production and mitigating stress responses (1, 38, 39). Additionally, research has found that oxidative stress may be a cause of anxiety, with elevated concentrations of pro-inflammatory cytokines in children and adolescents with anxiety (40). Exercise is linked to anti-inflammatory pathways and can either prevent or reduce the intensity of anxiety symptoms by regulating inflammatory and oxidative stress pathways (41, 42).

Aerobic exercise elevates fat-burning factors in children and adolescents while simultaneously lowering inflammation markers (43). This leads to an overall enhancement of physiological health, reduction in insulin resistance, decreased fat accumulation, and improved physical fitness (44, 45). Research consistently indicates that the hippocampus is crucial for managing stress levels, and individuals with depression tend to have a hippocampal volume that is approximately 5% smaller (9, 46, 47). Engaging in regular exercise has been linked to enhancements in cardiorespiratory fitness, which in turn correlates with an increase in the size of the hippocampus. Gujral’s study showed that 6 weeks of aerobic activity was adequate to boost hippocampal volume (37). Additionally, aerobic activity alters monoamine neurotransmitters, thereby increasing 5-HT and norepinephrine levels and lowering cortisol levels (48). These neurochemical changes help alleviate depression symptoms.

The intensity, session duration, frequency, and intervention period of aerobic exercise training may modulate its effects on depressive and anxiety symptoms. For anxiety, this study found that an aerobic exercise intervention at 60–89% of VO_2_ max, consisting of 60–75 min each session, 3–4 times a week for over 12 weeks, was most effective in improving children’s and adolescents’ anxiety. Clinical guidelines suggest three weekly aerobic sessions for depression symptom alleviation, a protocol strongly supported by our dose–response analysis showing maximal improvements at this frequency. Blumenthal’s research (46) also demonstrated that aerobic exercise exceeding 12 weeks was as effective as medication for alleviating anxiety symptoms. The American Academy of Pediatrics recommends a daily 60 min session of moderate-to-vigorous exercise to maintain both mental and physical well-being, aligning with our research findings (49). Notably, the subgroup analysis for anxiety within a 5–8 week period showed a non-significant effect, suggesting that within this shorter time frame, aerobic exercise may not have a significant impact on anxiety reduction. This finding is consistent with the hypothesis that anxiety requires long-term intervention to achieve significant improvement. Additionally, our study did not find significant effects of moderate-intensity (40–59% of VO_2_ max) exercise on anxiety. One factor could be the relatively small sample size of the moderate-intensity group, which may have limited the statistical power to detect significant effects. Another factor could be the specific characteristics of the study population, such as the baseline severity of anxiety symptoms, which may have influenced responsiveness to moderate-intensity exercise. Future research with larger sample sizes and more homogeneous populations is needed to further explore this relationship. For depression, our research found that moderate-to-high-intensity exercise (40–89% VO_2_ max), with each session lasting 60–75 min, conducted 3–4 times a week for 5–8 weeks, was the most effective in improving the depressive state of children and adolescents. Some studies have suggested that excessive exercise that leads to pain and injury may increase the risk of depression in children and adolescents (50, 51). For example, setting excessive exercise targets may cause additional stress and feelings of failure or exacerbate existing mental health symptoms in individuals with mental health disorders. This subgroup analysis shows that there is significant heterogeneity in the intervention effects of different exercise session durations, which may be attributed to the fact that studies adopting 15–30 min sessions often incorporated high-intensity exercises, where brief durations sufficed to trigger physiological changes conducive to mood regulation. Conversely, research with 60–75 min sessions predominantly featured lower-intensity, continuous aerobic activities. Additionally, some studies integrated warm-up and cool-down periods within specified durations, whereas others concentrated solely on core exercises, resulting in inconsistent effective exercise times and further complicating the assessment of intervention efficacy. Based on the findings of this research, not every aerobic exercise mode could improve all aspects of anxiety and depression performance in children and adolescents. Therefore, these outcomes highlight the need for exercise training programs to improve anxiety and depression in children and adolescents.

This study had some limitations. A notable concern was the variability in control group interventions across the included studies, which ranged from usual care and regular physical activity to no intervention. This diversity in control conditions introduced potential confounding factors, as different baseline activities and care models might have influenced the outcomes independently of aerobic exercise. Although all control groups lacked structured aerobic exercise programs, the heterogeneity in control interventions still complicated the direct comparison of the experimental group’s effectiveness, potentially affecting the internal validity of our findings. While we made adjustments for the heterogeneity among the studies when integrating their original findings, we remained unable to fully account for the variations present. In addition, the included studies exhibited variations in the types of exercise interventions, such as running, swimming, and cycling, and in the equipment used, such as treadmills and cycle ergometers. These factors were not uniformly controlled, potentially affecting the reliability and interpretability of the study results. In the future, objective criteria should be used to evaluate the indicators and minimize the risk of bias. In order to investigate potential modifiers of treatment effects, future research should record the study design and features in greater detail.

## 5 Conclusion

In conclusion, it is crucial to implement an optimal exercise intervention program to maximize the prevention or reduction of anxiety and depression among children and adolescents. The results indicate that aerobic exercise positively impacts anxiety and depression in youth. Additionally, for anxiety symptoms, we recommend a high-intensity exercise protocol (60–89% VO_2_ max, 60–75 min/session, 3–4 sessions/week), with an optimal intervention duration exceeding 12 weeks. For depression symptoms, we propose a moderate-to-high-intensity protocol (40–89% VO_2_ max, 60–75 min/session, 3–4 sessions/week) with a shorter optimal duration of 5–8 weeks.

## Data Availability

The original contributions presented in the study are included in the article/Supplementary material, further inquiries can be directed to the corresponding author.
