# Supplementary material for: Aerobic exercise strategies for anxiety and depression among children and adolescents: a systematic review and meta-analysis
Source: Front Public Health. 2025 Jul 1;13:1555029. doi: 10.3389/fpubh.2025.1555029 (PMC12261993; doi:10.3389/fpubh.2025.1555029)
Supplement: Supplementary file 1 [file Data_Sheet_1.docx]

**File S1 Search strategy**

| **Databases** | **Keywords** |
| --- | --- |
| PubMed | ("youth"[Title/Abstract] OR "adolescent"[Title/Abstract] OR "teenager"[Title/Abstract] OR "child"[Title/Abstract] OR "children"[Title/Abstract] OR "student"[Title/Abstract]) AND ("physical activity"[Title/Abstract] OR "physical exercise"[Title/Abstract] OR "sport movement"[Title/Abstract] OR "sport"[Title/Abstract] OR "athletic sports"[Title/Abstract] OR "aerobic exercise"[Title/Abstract] OR "aerobic training"[Title/Abstract] OR "fitness game"[Title/Abstract]) AND ("depression"[Title/Abstract] OR "depressive"[Title/Abstract] OR "anxiety"[Title/Abstract] OR "mental health"[Title/Abstract] OR "emotional symptom"[Title/Abstract] OR "anxious"[Title/Abstract]) |
| Scopus | ( TITLE-ABS (youth OR adolescent OR teenager OR child OR children OR student) AND ( TITLE-ABS ("physical activity" OR "physical exercise" OR "sport movement" OR sport OR motor OR "athletic sports" OR "aerobic exercise" OR "aerobic training" OR"fitness game") AND TITLE-ABS (depression OR depressive OR anxiety OR "'mental health" OR "emotional symptom" OR anxious ) ) |
| Web of science | ((AB=(youth OR youths OR adolescent OR adolescents OR teenager OR teenagers OR child OR children OR student)) AND AB=(“physical activity” OR “physical exercise” OR “sport movement” OR sport OR motor OR “athletic sports” OR “aerobic exercise” OR “aerobic training” OR “fitness game”)) AND TS=(depression OR depressive OR anxiety OR “‘mental health” OR “emotional symptom” OR anxious) |
