# Supplementary material for: Aerobic exercise strategies for anxiety and depression among children and adolescents: a systematic review and meta-analysis
Source: Front Public Health. 2025 Jul 1;13:1555029. doi: 10.3389/fpubh.2025.1555029 (PMC12261993; doi:10.3389/fpubh.2025.1555029)
Supplement: Supplementary file 2 [file Data_Sheet_2.docx]

**File S2. Quality evaluation**

| **Study** | **Each Question** | | | | | | | | | | | **Total score** |
| --- | --- | --- | --- | --- | --- | --- | --- | --- | --- | --- | --- | --- |
|  | 1 | 2 | 3 | 4 | 5 | 6 | 7 | 8 | 9 | 10 | 11 |  |
| Bao 2015 | Y | Y | N | Y | Y | N | N | Y | Y | Y | Y | 8 |
| Dabidy 2011 | Y | N | N | Y | N | N | N | Y | Y | Y | Y | 6 |
| Fidelix 2019 | Y | N | Y | Y | N | N | N | Y | Y | Y | Y | 7 |
| Goldfield 2015 | Y | Y | Y | Y | N | N | Y | Y | Y | Y | Y | 9 |
| Jelalian 2011 | Y | Y | Y | Y | N | N | Y | Y | Y | Y | Y | 9 |
| Jeong 2005 | Y | N | Y | Y | N | N | N | Y | Y | Y | Y | 7 |
| Luo 2021 | Y | N | Y | Y | N | N | Y | Y | Y | NA | Y | 7 |
| Melnyk 2009 | Y | N | N | Y | N | N | N | Y | Y | Y | Y | 6 |
| Nazari 2020 | Y | Y | N | Y | Y | N | Y | Y | Y | Y | Y | 9 |
| Petty 2009 | Y | Y | N | Y | Y | N | N | Y | Y | Y | Y | 8 |
| Philippot 2022 | Y | Y | N | Y | Y | N | N | Y | Y | Y | Y | 8 |
| Roh 2018 | Y | N | N | Y | N | N | N | Y | Y | Y | Y | 6 |
| Romero 2020 | Y | Y | Y | Y | N | NA | Y | Y | Y | Y | Y | 9 |
| Roth 1987 | Y | N | N | Y | N | N | N | Y | Y | NA | Y | 5 |
| Silva 2020 | Y | Y | Y | Y | N | N | N | Y | Y | Y | Y | 8 |
| Talakoub 2012 | Y | Y | N | Y | Y | N | N | Y | Y | Y | Y | 8 |
| Wagener 2012 | Y | Y | Y | Y | N | N | N | Y | Y | Y | Y | 8 |
| Weintraub 2008 | Y | NA | N | Y | N | N | N | Y | Y | NA | Y | 5 |
| Williams 2019 | Y | Y | N | Y | N | N | N | Y | Y | Y | Y | 7 |

**Key: Y= Yes; N= Not, NA=Not appropriate**

**Question codes:**

**1. Was eligibility criteria specified?**

**2. Were all subjects randomly allocated?**

**3. Were allocations concealed?**

**4. Were the groups similar at baseline?**

**5. Was there blinding of all participants?**

**6. Was there blinding of all therapists?**

**7. Was there blinding of all assessors?**

**8. Was there measures of at least one key outcome for more than 85% of the subjects initially allocated to groups?**

**9. Did all subjects for whom outcome measures were available receive the treatment or control condition as allocated or, where this was not the case, data for at least one key outcome was analyzed by “intention to treat”**

**10. Were the results of between group statistical comparisons reported for at least one key outcome?**

**11. Did the study have both point measures and measures of variability for at least one key outcome.**
